# Inferior vestibular neuritis: 3 cases with clinical features of acute vestibular neuritis, normal calorics but indications of saccular failure

**DOI:** 10.1186/1471-2377-6-45

**Published:** 2006-12-14

**Authors:** Per Monstad, Siri Økstad, Åse Mygland

**Affiliations:** 1 Department of Neurology, Sørlandet Sykehus, Kristiansand, Norway; 2 Department of otolaryngology, Sørlandet Sykehus, Kristiansand, Norway

## Abstract

**Background:**

Vestibular neuritis (VN) is commonly diagnosed by demonstration of unilateral vestibular failure, as unilateral loss of caloric response. As this test reflects the function of the superior part of the vestibular nerve only, cases of pure inferior nerve neuritis will be lost.

**Case presentations:**

We describe three patients with symptoms suggestive of VN, but normal calorics. All 3 had unilateral loss of vestibular evoked myogenic potential. A slight, asymptomatic position dependent nystagmus, with the pathological ear down, was observed.

**Conclusion:**

We believe that these patients suffer from pure inferior nerve vestibular neuritis.

## Background

The diagnosis of acute vestibular neuritis requires objective findings indicating complete or partial unilateral loss of vestibular function. In ordinary clinical practice vestibular integrity is tested by caloric irrigation and the horizontal head impulse test [1]. Both of these procedures test the horizontal semicircular canal. Patients who present with similar symptoms but normal caloric test results are commonly assumed to suffer from a lesion of the central nervous system [2].

Recently, Halmagyi et al [3] presented two cases of acute vertigo with normal calorics but with pathological vestibular evoked myogenic potential (VEMP) and other findings indicating a lesion of the inferior part of the vestibular nerve, with sparing of the superior vestibular nerve. Cases of selective loss of inferior vestibular nerve will not be identified as such if vestibular tests are restricted to calorics. The introduction of the Vestibular Evoked Myogenic Potential allows examination of saccular function, innervated by the inferior part of the nerve. In our hospital, VEMP has been part of the routine work-up of suspected vestibular neuritis since mid 2004.

We perform the test according to Colebatch & al [4], using rarefaction click stimuli at a sound intensity of 90 dB above normal hearing level.

We report three patients with probable "inferior vestibular neuritis".

## Case presentations

### Pat 1

A previously healthy 72 years old female presented with rotatory vertigo associated with nausea and vomiting. The symptoms had started five days earlier as short lasting vertigo attacks, her symptoms had been continuous for three days. Clinical examination showed BP 200/95. Otoscopy was normal No nystagmus in a neutral position, and no pathological findings by standard clinical neurological examination was found. The next day the patient was still nauseated and described continuous vertigo, with a slight improvement. Hallpikes manoeuvre did not evoke nystagmus or exacerbation of her vertigo.

Horizontal head impulse test, headshaking test with Frenzel goggles and subjective visual vertical [5] were normal. ENG showed no nystagmus when the head was in neutral position (30 degrees upward tilt from supine), and a weak right beating nystagmus (slow phase velocity 15–19 degrees per second), but no symptoms, when turned to the left.

Calorics were normal. VEMP showed a low amplitude response on the right, and loss of response on the left. Audiogram showed slight presbyacusis; normal hearing up to 3 kHz.

CT scan and MRI was normal.

On follow up at four weeks later her symptoms had improved but not disappeared completely. ENG still showed right beating nystagmus in the left-rotated position, and still normal calorics. A new VEMP was recorded. This time the test was noted to be unpleasant for the patient, and cooperation was suboptimal. Absent responses on both sides were found.

### Pat 2

77 years old female, previously healthy except for a short period of vertigo two years earlier. She presented with a two day long history of vertigo, nausea and vomiting, still nauseated and with inability to walk. Standard clinical exam showed no limb ataxia or other obvious pathological findings. Removal of fixation with the use of Barthels goggles revealed a counterclockwise nystagmus (with respect to observer), combined with nystagmoid jerks to the right. This nystagmus was completely suppressed by fixation. VEMP was normal on the right side, absent on the left. ENG was performed two weeks later. A weak nystagmus, fast beat to the right, was found, only when left ear was lower (slow phase velocity 10–14 degrees per second). Calorics demonstrated no significant asymmetry. MRI was normal except for minor white matter changes. Otoscopy and audiometry were normal. She made a good recovery but was still complaining of intermittent vertigo at follow up, two weeks after admission.

### Pat 3

**Figure 1:**
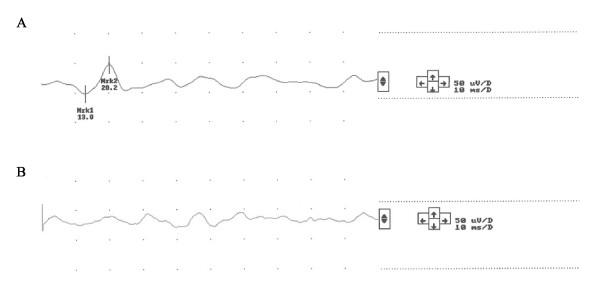
VEMP after right ear clicks, recorded ipsilaterally (1A) and vice versa for left side stimulation (1B). Pat 3.

**Figure 2:**
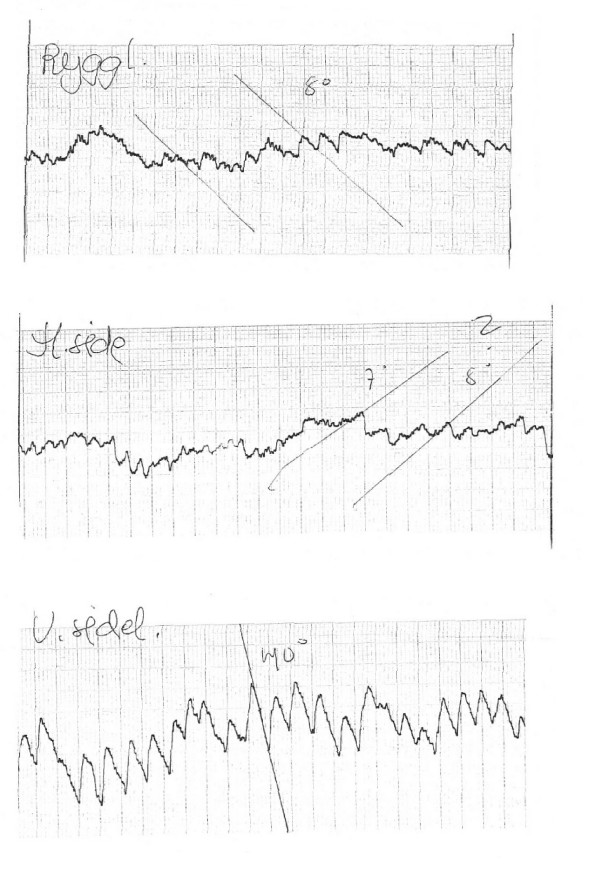
ENG from pat 3, showing recordings in supine pos (upper curve), right and left side (middle and lower curves). A distinct an non-decaying nystagmus was seen when turned to the left.

A 64 yrs old female presented with vertigo, nausea and headache of two days duration. On admission she had no nystagmus, and normal clinical exam. including otoscopy. Audiometry and MRI was normal. VEMP showed attenuated/scarcely identifiable response on the left side (Figure 1A and 1B). ENG showed slight nystagmus (7–8 degrees per second) in neutral (supine) position, which increased markedly, to a slow phase velocity of 40 degrees per second, on left rotation of the head, beating to the right (Figure 2). She reported no symptoms during these positional tests. Calorics showed no significant asymmetry.

Her condition improved but she had still symptoms at follow up two weeks after onset. On follow up five months later she reported some vertigo in episodes of 1–2 days duration, but was free of symptoms most of the time. ENG showed only slight spontaneous nystagmus is left position, and calorics was normal and symmetrical. VEMP was still absent on the left side, and normal on the right.

## Conclusion

All three patients presented with symptoms suggestive of acute vestibulopathy, vestibular neuritis. The calorics were normal, however. Hypofunction of the lateral canal, as shown by a loss or decrease of caloric response, is a "sine qua non" of vestibular neuritis, as commonly defined.

Most patients with VN have loss of function in the superior portion of the nerve, exclusively, while perhaps one third have loss of function in the inferior nerve as well. [6-8]. The paucity of reports of VN with selective lesion of the inferior nerve has been explained by the shorter and wider ossesous canal of the inferior nerve compared to the superior branch [9]. This would make the inferior nerve less susceptible to entrapment during inflammatory (HSV-induced?) swelling. VN with selective lesion of the inferior nerve (iVN) is difficult to diagnose, owing to the lack of clinical tests for posterior canal or saccular function. It is possible that iVN will be wrongly classified as minor stroke or, more likely, remain undiagnosed.

Although head impulse test with scleral coil technique can identify loss of function of the posterior canal, this advanced technique is out of reach for most hospitals who treat acute vertigo patients.

VEMP is a test of saccular function. It can easily be performed, using ordinary equipment for evoked potentials. Halmagyi & al has presented two patients with probable inferior nerve VN [3], one with simultaneous hearing loss and one with normal hearing. Patient no 1 in their paper has the same characteristics as our patients, with one exception: Their patient had no positional nystagmus. Iwasaki & al [10] reported a series of 811 patients who were examined by calorics and VEMP. 40 patients had abnormal VEMP despite normal calorics. In 8 patients no established diagnosis could be made. Although some of their patients might have suffered from vestibular migraine, a disorder which can present with these findings [11], a neuritis of the inferior vestibular nerve was assumed to be the most probable explanation.

We believe that our three patient suffer from inferior nerve VN (iVN). Cerebrovascular lesions can not be completely ruled out in these cases. Even patients with symptoms and signs of classical vestibular neuritis might suffer from CNS lesions, mainly vascular, in up to 15 % of cases [12,13]. Pat 1 had hypertension, and developed loss of VEMP on both sides later, after initially showing only unilateral loss. VEMP is an ipsilateral reflex, and cerebrovascular mechanisms for bilateral loss of VEMP are unlikely in cases with no other clinical findings indicative of a brainstem disorder. Pat 2 had some minor white matter lesions on MRI. No lesions were found in the brainstem in these patients. Diffusion-weighted MRI was not available and this might reduce the sensitivity of MRI in the diagnosis of pontine lacunar strokes. Absent or reduced unilateral VEMP has been described in patients with MRI signs of lower brainstem lesions, whether cerebrovascular or inflammatory [14]. However, multiple sclerosis patients might suffer from loss of VEMP without obvious lesions in the brainstem that explain this finding [15], and this might be the case in cerebrovascular accidents as well. Loss of VEMP will probably demand a pontine or medullary lesion [14]. Our patients had no other findings or symptoms that suggest such CNS lesions. The symptoms, as well as the symptomatic recovery on follow up are suggestive of a peripheral vestibular lesion. VEMP is extremely sensitive to conductive hearing loss, even subclinical conductive hearing impairment might result in loss of VEMP. Our patients had normal audiograms, and no middle ear pathology. Under these circumstances, VEMP is a fairly robust test of saccular function.

Loss of VEMPs might be seen even in healthy older persons [16,17]. The unilateral loss observed in our patients must be presumed to be pathological, though.

The three common factors in our cases are: Normal calorics, unilateral loss of VEMP, and slight, clinically silent, positional nystagmus with the pathological ear down, beating away from the lesioned ear. This positional nystagmus was not obvious by ordinary inspection without use of Frenzels goggles, and was only detected by ENG. The limitations of conventional ENG prevent complete description of the eye movements, whether a rotatory component was present is unknown. Benign paroxysmal positioning vertigo caused by canalolithiasis is a common sequela of VN, but a such mechanism cannot explain our observation: The position dependent symptoms were mild or absent during test procedures, and, more important: The nystagmus did not end during the two minute observation time: There was no time constant, or a very long one. Cupulolithiasis of the horizontal canal will result in apogeotropic nystagmus, which is most pronounced with the healthy ear down. Although cupulolithiasis is a possible explanation for the observed positional nystagmus in our cases, we find this very unlikely: The observed nystagmus was mainly (pat 2) or exclusively (pat 1 and 2) present when the pathological ear (judged by absence of VEMP) was down. To explain the findings by BPPV, a contralateral horizontal canal cupulolithiasis would have to be present in all three cases. The absence of apogeotropic nystagmus with the right ear down in pat 1 and 2 and nystagmus within normal limits (6–8 degrees) in pat 3 also make this an unlikely assumption.

Positional nystagmus which is not caused by BPPV is generally assumed to result from CNS lesions, most often cerebellar pathology.

Although rarely mentioned in the literature, positional nystagmus is sometimes seen after ordinary VN [18]. The cause of head position dependent nystagmus in our patients is unknown. Current knowledge of saccular physiology is rather limited, and can not explain this finding. Both utriculi and sacculi respond to static tilt. Saccular stimulation has probably no time constant, as opposed to stimulation of the semicircular canals. The constant (non-decaying) position – dependent nystagmus seen in our patients might be produced by lack of the expected saccular input. How this transforms into positional nystagmus is difficult to understand, however. Saccular dysfunction causes an asymmetric failure of the velocity-storage mechanism [19]. Loss of otolithic input from the utricle, after surgical lesion of the utricular nerve in cats, has been shown to result in positional nystagmus with the lesioned ear down [20]. Our patients had probably normal utricular function, as this organ is innervated by the superior vestibular nerve. Only pat 1 was tested by subjective visual vertical, a test of utricular function, this was found to be normal. A similar experiment with lesion of the sacculus has not been done, to our knowledge.

A weak but persistent positional nystagmus (mostly with a slow phase velocity of less than 6–8 degrees per second) is common even in healthy individuals, with a prevalence of persistent nystagmus in at least one head position of up to 51% [21]. The velocity of the slow phase in our patients were significantly higher, and can not be explained as a normal phenomenon. Thus, positional nystagmus that cannot be explained by BPPV should not be considered a sign of CNS pathology only.

Larger studies will be necessary to validate these findings.

## Abbreviations

VEMP: Vestibular evoked myogenic potential. VN: Vestibular neuritis. BPPV Benign paroxysmal positioning nystagmus. IVN: inferior nerve vestibular neuritis. ENG:Electronystagmography.

## Competing interests

The author(s) declare that they have no competing interests.

## Authors' contributions

PM and SØ examined the patients. PM made the initial draft of the manuscript. AM participated in the scientific discussion and revised the final manuscript in cooperation with PM and SØ. All authors read and approved the final manuscript.

## Pre-publication history

The pre-publication history for this paper can be accessed here:
